# Valuing health across groups: a cross-sectional population-based willingness-to-pay survey in Bhutan

**DOI:** 10.1136/bmjgh-2025-019098

**Published:** 2025-08-21

**Authors:** Ying Yao, Md. Mizanur Rahman, Pempa, Yot Teerawattananon, Ryota Nakamura

**Affiliations:** 1 Faculty of Economics, Keio University, Tokyo, Japan; 2 Hitotsubashi Institute for Advanced Study, Hitotsubashi University, Tokyo, Japan; 3 Department of Health Services, Ministry of Health, Thimphu, Bhutan; 4 Health Intervention and Technology Assessment Program, Ministry of Public Health, Nonthaburi, Thailand; 5 Saw Swee Hock School of Public Health, National University of Singapore, Singapore; 6 Department of Global Health and Development, London School of Hygiene & Tropical Medicine, London, UK

**Keywords:** Health economics, Health policy, Cross-sectional survey

## Abstract

**Introduction:**

Context-specific cost-effectiveness thresholds (CETs) informed by societal willingness to pay (WTP) are crucial for healthcare resource allocation in low- and middle-income countries. This study investigated WTP for health per quality-adjusted life year (QALY) in Bhutan.

**Methods:**

A WTP survey was conducted alongside the 2023 National Health Survey in Bhutan, sampling 1869 households. Using contingent valuation, respondents assigned monetary values to three hypothetical scenarios: 1 year of cancer symptom-free life, 1 year of perfect health and 5 years of perfect health. We used generalised linear regression to estimate WTP, controlling for demographic, socioeconomic and health-related factors, as well as elicitation methods. Multilevel analyses examined WTP variations within and between districts.

**Results:**

WTP estimates were 76 836 Bhutanese ngultrum (BTN) (0.26 times gross domestic product (GDP) per capita; 95% CI: 71 397 to 82 275) for a year without cancer symptoms, 104 381 BTN (0.35 times GDP per capita; 95% CI: 96 405 to 112 357) for 1 QALY and 235 237 BTN (0.78 times GDP per capita; 95% CI: 218 674 to 251 800) for 5 QALYs. WTP variations were driven by within-district individual characteristics, particularly income and education, rather than between-district differences.

**Conclusion:**

Minimal between-district WTP variations support a national-level CET for Bhutan. However, WTP-based CETs would be biased upward by wealthier and more educated groups. This bias could justify expensive technologies that strain public resources in Bhutan’s government-funded healthcare system.

**WHAT IS ALREADY KNOWN ON THIS TOPIC**

- Limited evidence exists on the population’s willingness to pay (WTP) for health interventions in Bhutan.

**WHAT THIS STUDY ADDS**

- This study provides the first population-based estimates of WTP for health in Bhutan, offering insights into the consumption value of population health and a cost-effectiveness threshold for health resource allocation.

**HOW THIS STUDY MIGHT AFFECT RESEARCH, PRACTICE OR POLICY**

- The findings can guide policymakers in setting more context-specific health financing strategies and inform the design of sustainable health programmes aligned with public preferences.

## Introduction

 Healthcare systems worldwide face challenges from constrained budgets and increasing expenditures fuelled by ageing populations, the rise of chronic diseases and emerging medical technologies. To ensure long-term affordability, public healthcare systems require interventions to improve performance and maintain adequate financing.^1^ One key approach to healthcare resource allocation is prioritising healthcare programmes based on the consumption value of population health, which can be informed by willingness-to-pay (WTP) studies.

In these evaluations, WTP per additional quality-adjusted life year (QALY) gained is often used to inform cost-effectiveness thresholds (CETs). These thresholds have various applications in healthcare decision-making. In the public sector, CETs help shape benefit package design and guide resource allocation decisions.^2^ In the private sector, WTP evidence can inform pricing strategies, insurance premiums and copayment structures.^3^ Many high-income and upper-middle-income countries, such as the UK, Australia, the USA, some European nations and Thailand, have researched WTP-based CETs to build evidence that supports informed healthcare decision-making.^4^

The growing interest in CET reflects a broader shift towards context-specific healthcare decision-making. While the WHO’s uniform threshold of 1–3 times gross domestic product (GDP) per capita has been widely used, this one-size-fits-all approach may not fully reflect diverse societal preferences. Understanding how WTP for health varies by demographic, socioeconomic and regional factors is crucial for developing healthcare policies. For instance, if research reveals substantial regional variations in WTP for health gains, policymakers could consider geographically tailored CETs that better reflect local priorities and resource constraints.

This need for context-specific WTP data is particularly important in low- and middle-income countries (LMICs), where intra-country disparities in health outcomes and resource availability require locally tailored resource allocation decisions. Despite this pressing need, many LMICs lack comprehensive WTP studies, creating a critical knowledge gap that hinders evidence-based decision-making.^5^ Bhutan, an LMIC in South Asia, exemplifies these challenges, as its policymakers face difficult healthcare financing without local data on societal preferences for health spending.^6^

With a GDP per capita of approximately US$3833,^7^ Bhutan operates a tax-financed health system that covers the entire population. This system ensures that 90% of residents live within 2 hours of a healthcare facility and provides free healthcare services at all levels, from primary healthcare to advanced treatments, including services at designated hospitals abroad.^8^ However, Bhutan’s healthcare system is under increasing pressure due to growing demands for advanced treatments and declining external funding. In 2020, the government provided 73.4% of the current health expenditure, households contributed 15.4% and non-governmental organisations and donors represented 7.3%.^9^ These pressures require the optimisation of resource allocation within budget constraints to maintain the healthcare system.

This study aims to provide empirical evidence to inform Bhutan’s healthcare financing decisions by estimating WTP for healthcare among the Bhutanese population. By examining WTP determinants, we provide insights into the monetary value placed on health improvements in Bhutan. These insights are crucial for establishing a contextually appropriate CET in Bhutan and enhancing the comprehension of how resource allocation decisions can be effectively adapted to local contexts in LMICs.

## Methods

### Survey and modules

**Figure 1:**
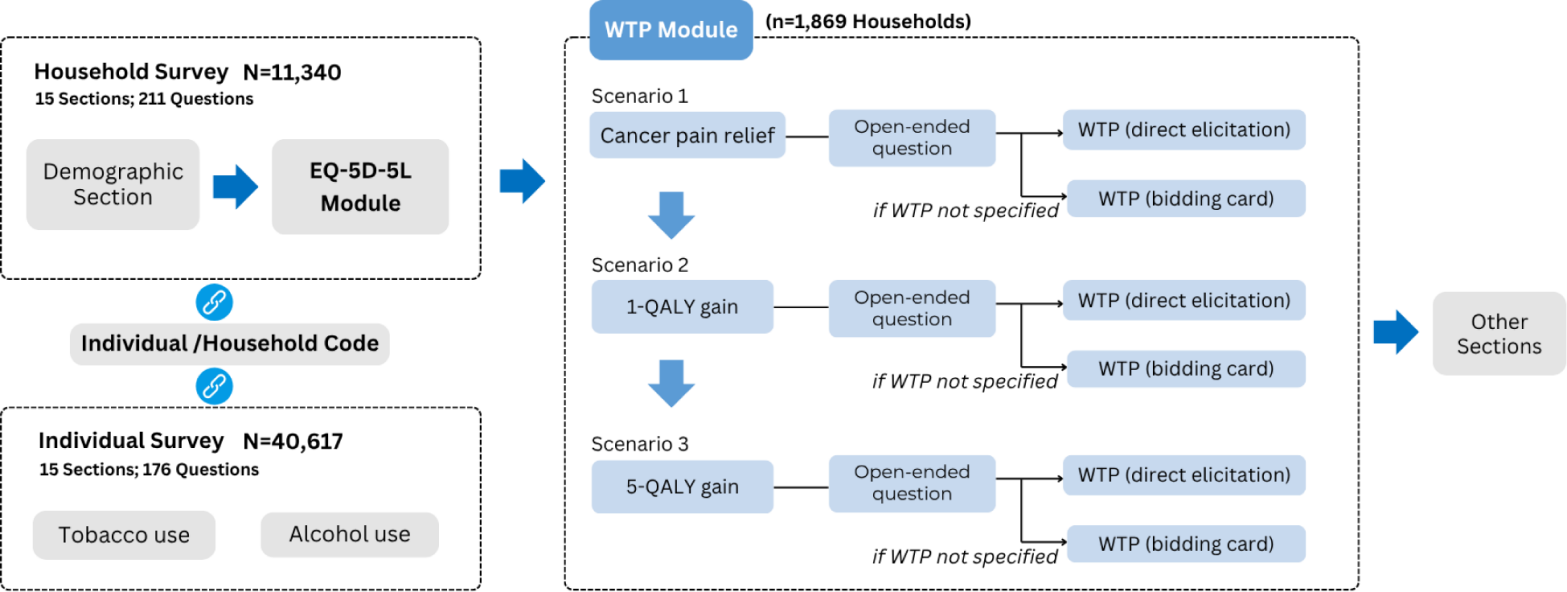
Module Structure and design of the 2023 National Health Survey in Bhutan. The figure shows only the modules and survey sections relevant to our analysis. The survey covered 20 districts and four municipalities across Bhutan. It is supported by the Royal Government of Bhutan, the WHO, the United Nations Population Fund, UNICEF, the World Food Programme, Hitotsubashi University and the Asian Development Bank. EQ-5D-5L, EuroQol 5-Dimension 5-Level; QALY, quality-adjusted life-year; WTP, willingness to pay.

This study used the latest data from the 2023 National Health Survey in Bhutan, conducted from May to July 2023 by the Ministry of Health and the National Statistics Bureau. The survey, launched in 1984 and conducted every 10 years, monitors trends in the health status of Bhutanese nationals and permanent residents. The 2023 edition included four main modules: household, individual, women and children and physical measurement and biochemistry. Two new modules were added to the household survey: the EuroQol 5-Dimension 5-Level (EQ-5D-5L), a standard instrument for measuring health-related quality of life (HRQoL) and the WTP module assessing societal preference for health (figure 1). These new modules enable the analysis of relationships between WTP outcomes and various factors, including demographic, socioeconomic, behavioural, health status and district-level characteristics.

The survey questionnaire, developed in English on the Survey Solutions platform, was administered via face-to-face interviews by trained interviewers. Interviews were conducted in local dialects, as appropriate. Multilevel supervision ensured data quality, with team supervisors reviewing responses, verifying data and revisiting or calling back households to correct inaccuracies. An independent consultant and the technical working group performed frequent data checks, unannounced monitoring visits and spot checks.

### Data and sampling

A stratified two-stage sampling method was employed to obtain a representative sample from Bhutan’s population of 0.76 million. Neighbourhoods or villages served as primary sampling units (PSUs) for urban and rural areas, respectively. Households within each PSU were selected using circular systematic sampling to ensure even distribution. In total, 990 PSUs were identified, with 12 households selected per PSU. From each PSU, two households were chosen for the WTP module, with one individual from each responding. Data were collected from 11 340 households and 40 617 household members, with 1869 households participating in the WTP module.

### WTP questionnaire and scenarios

The WTP questionnaire followed the EQ-5D-5L module, which was used to help participants understand the concept of perfect health. The WTP section employed the contingent valuation method, where respondents were asked to assign monetary values to a hypothetical improvement in health status across three scenarios. The research team piloted and validated the questionnaire at the study sites in January 2023. The final instrument presented participants with three consecutive healthcare payment scenarios.

- *Scenario 1*: imagine that you are suffering from *cancer* that immediately threatens your life. Now, please assume that we have a new drug that will provide relief from cancer symptoms or side effects from cancer treatments for a duration of 1 year. If the national health system does not fund the new drug, you must pay by yourself.
- *Scenario 2*: imagine that you are stricken with a *serious illness* that immediately threatens your life. Now, please assume that we have a new treatment that allows you to return to a 1year perfect health state (no pain, perfect mobility, total autonomy for personal care and daily activities and absence of anxiety or depression). Imagine that the effect lasts only while you are taking it, and it has no side effects. If the national health system does not fund the new treatment, you must pay by yourself.
- *Scenario 3*: imagine that you are stricken with a *serious illness* that immediately threatens your life. Now, please assume that we have a new treatment that allows you to return to a 5-year perfect health state (no pain, perfect mobility, total autonomy for personal care and daily activities and absence of anxiety or depression). Imagine that the effect lasts only while you are taking it, and it has no side effects. If the national health system does not fund the new treatment, you must pay by yourself.

All scenarios required participants to pay out-of-pocket because the national health system would not fund the treatments. The first scenario introduced cancer as an example of a life-threatening illness since Bhutanese people widely understand its severe outcomes, including health deterioration, pain and suffering. This generic cancer scenario helped participants grasp the concept of serious illnesses. Participants were asked to imagine they had been diagnosed with terminal cancer. They were then presented with a hypothetical new drug that could relieve all symptoms of cancer for 1 year, although it would not prevent their death at the end of that period. The second and third scenarios aimed to estimate the value of a QALY by presenting a life-threatening disease with a new treatment option. The second scenario offered treatment providing 1 year of perfect health (1 QALY), while the third offered 5 years (5 QALYs). In both cases, health would revert to the pretreatment state after the specified period, with death presented as the outcome.

In all scenarios, we followed a standardised process. Participants were first asked if they would be willing to pay for the treatment. Those who answered ‘No’ were asked to provide their reasons. If they answered ‘Yes,’ participants were asked to indicate the maximum amount they were willing to pay out-of-pocket through an open-ended question, ‘How much are you willing to pay for the new drug?’ They could state their maximum WTP as either monthly payments or a 1-year lump sum. When participants could not provide a direct amount, enumerators used a structured bidding process with a predefined payment card to help determine their maximum WTP. The complete questionnaire, including the payment card, is included in the online supplemental materials.

Bidding values ranged from 30 000 Bhutanese Ngultrum (BTN) to 90 000 BTN per month. Initial bids were set at 30 000 BTN for the cancer and 1-QALY scenarios and 70 000 BTN for the 5-QALY scenario. These starting values were equivalent to 1–3 times Bhutan’s GDP per capita in 2022 (301 289 BTN).^7^ The bidding process followed a two-directional approach. If respondents accepted the initial bid, subsequent bids increased until they responded ‘No,’ with the highest accepted amount recorded as their WTP. If they rejected the initial bid, progressively lower amounts were offered until they responded ‘Yes,’ at which point that amount was recorded as their WTP. For both elicitation methods, monthly figures were subsequently converted into annual amounts. Following the WTP questions, participants were asked about the primary sources of financing for these out-of-pocket payments. Those unwilling to pay were asked to explain their reasons for refusal.

Since the questionnaire did not explicitly record the elicitation method, respondents were classified as bidding method users if their recorded WTP matched the predetermined amounts on the payment card. To minimise misclassification due to random coincidence, we required matches in both the first and second scenarios for classification. While this approach may not capture all instances of bidding method use, respondents who consistently selected predetermined values across multiple scenarios were likely guided through the structured bidding process.

### Statistical analyses

Descriptive analyses were used to examine participants’ characteristics across multiple domains: demographic factors (age, sex, marital status), socioeconomic (educational levels, working status, residential area, household size, annual income) and health status and behaviours (tobacco and alcohol consumption). Health status was assessed through HRQoL using the EQ-5D-5L questionnaire, with utility scores derived using the Indian value set due to the absence of Bhutan-specific values.^10^ These calculations were performed using the ‘eq5d’ R package (V.0.15.4).^11^ We also compared characteristics between WTP survey participants and non-participants using Pearson’s tests for categorical variables and Kruskal-Wallis tests for continuous variables. In addition, standardised mean differences were calculated to quantify the magnitude of observed group differences. The same approach was applied to compare users of direct and bidding methods, as systematic differences in participant characteristics could influence WTP estimates.

For the main analysis, WTP values were stratified by elicitation method and summarised using means with 95% CIs. The Mann-Whitney U test was used to assess whether WTP distributions differed significantly between groups. WTP values were estimated using generalised linear regression models. The regression models included individual-level covariates, district fixed effects to control for unobserved geographic variation and a dummy variable for elicitation method (1 for bidding, 0 for direct elicitation) to adjust for differences between the two approaches. Due to the skewed distribution of WTP data, a Gamma regression with the log-link function was applied. This approach was chosen over log transformation to avoid biased predictions when reversing the transformation.^12^ A sensitivity analysis excluded respondents who expressed no WTP values in any of the three scenarios or demonstrated inconsistent valuations (eg, valuing 1 year of perfect health higher than 5 years).

To identify the relative contribution of individual and geographical factors in explaining WTP variations, we conducted a complementary analysis using multilevel regression models.^13 14^ Understanding these relative contributions would inform whether policy interventions should prioritise district-level healthcare system improvements or individual support programmes. This approach accounted for the hierarchical structure of our data, where individuals are nested within districts, potentially sharing unobserved characteristics that could affect their WTP.

Multilevel modelling enabled simultaneous examination of individual-level and district-level effects on WTP, quantifying the proportion of WTP variation attributable to differences between versus within districts. We implemented a two-level model with individuals at level one and districts at level two, incorporating random effects (capturing district-level variability) and fixed effects (capturing specific WTP predictors). The intraclass correlation coefficient (ICC) quantified the relative contributions of district vs individual variations. We assessed how much individual characteristics explained the observed WTP variations by comparing models with district effects only (null model) and with both district and individual characteristics (full model).

### Patient and public involvement

Patients or the public were not involved in the design, implementation or reporting of this study.

## Results

### Characteristics of survey participants

Of the 11 340 households surveyed, 1869 (16.5%) participated in the WTP module. Among these participants, 46 (2.5%) reported no WTP out-of-pocket for any of the three scenarios. In addition, 16 responses (0.9%) were flagged as inconsistent, as respondents valued 1 year of perfect health higher than 5 years.

Demographically, nearly two-thirds of the sample were women; over 75% were married or living together, while 15% had never married, and 10% were divorced, separated or widowed. Socioeconomically, more than half were employed, primarily in agriculture (40%), services (20%) or professional roles (12%). Around 63% of the sample lived in urban areas, and the average annual income was around 265 100 BTN, with a large SD of 1 093 500 BTN. In terms of health behaviours, over 90% were non-smokers, and 62% had not consumed alcohol in the past 12 months. Frequent drinkers (1–2 days per week to daily) made up 18%, while occasional drinkers (less than once a month to 1–3 days per month) accounted for 20%. As for health status, the population exhibited near-perfect health, with an average EQ-5D-5L utility score of 0.9.

Statistical differences between WTP participants and non-participants were observed across several demographic and health-related factors (table 1). WTP participants were on average 1 year younger and had higher education levels, larger households and greater annual incomes. They were also more likely to drink occasionally and less likely to be either non-drinkers or frequent drinkers. Regarding health, approximately 57% of WTP participants reported perfect health (ie, utility scores equal to one) and had higher overall EQ-5D-5L utility scores. Despite these statistical differences, standardised mean differences for all covariates were below 0.1, suggesting that the magnitude of these differences was negligible (online supplemental figure S1).

**Table 1:** Characteristics of survey participants

	WTP non-participants	WTP participants	All participants	Difference test
	(n=9471)	(n=1869)	(n=11 340)	
Age	40.9±13.8	39.7±13.4	40.7±13.7	<0.001
Sex				
Male	3414 (36.0%)	640 (34.2%)	4054 (35.7%)	0.137
Female	6057 (64.0%)	1229 (65.8%)	7286 (64.3%)	
Marital status				
Never married	1367 (14.4%)	276 (14.8%)	1643 (14.5%)	0.232
Married/living together	7076 (74.7%)	1415 (75.7%)	8491 (74.9%)	
Divorced/separated/widowed	1028 (10.9%)	178 (9.5%)	1206 (10.6%)	
Educational level				
No education	3928 (41.5%)	665 (35.6%)	4593 (40.5%)	<0.001
Primary or below	1333 (14.1%)	299 (16.0%)	1632 (14.4%)	
Secondary or equivalent	3492 (36.9%)	740 (39.6%)	4232 (37.3%)	
Tertiary	716 (7.6%)	165 (8.8%)	881 (7.8%)	
Working status				
Not working	4293 (45.3%)	816 (43.7%)	5109 (45.1%)	0.185
Currently working	5178 (54.7%)	1053 (56.3%)	6231 (54.9%)	
Residential area				
Urban	3519 (37.2%)	699 (37.4%)	4218 (37.2%)	0.842
Rural	5952 (62.8%)	1170 (62.6%)	7122 (62.8%)	
Household size	3.5±1.6	3.6±1.6	3.5±1.6	0.009
Annual income (1000 BTN)	267.3±1190.8	254.3±265.2	265.1±1093.5	0.014
Smoking status				
Current smoker	861 (9.1%)	180 (9.6%)	1041 (9.2%)	0.697
Ever smoker	1377 (14.5%)	277 (14.8%)	1654 (14.6%)	
Non-smoker	7229 (76.4%)	1411 (75.5%)	8640 (76.2%)	
Drinking frequency				
Not drinking	5917 (62.5%)	1153 (61.7%)	7070 (62.4%)	0.042
Frequent drinking	1685 (17.8%)	304 (16.3%)	1989 (17.5%)	
Occasional drinking	1865 (19.7%)	411 (22.0%)	2276 (20.1%)	
EQ-5D-5L Utility Score	0.9±0.1	1.0±0.1	0.9±0.1	0.009
Imperfect health	4361 (46.0%)	796 (42.6%)	5157 (45.5%)	0.006
Perfect health	5110 (54.0%)	1073 (57.4%)	6183 (54.5%)	

Note: Mean±SD is reported for continuous variables. N (%) is reported for factor variables. Kruskal-Wallis test for continuous variables. Pearson’s test for factor variables.

BTN, Bhutanese Ngultrum; WTP, willingness to pay.

Among WTP participants, 1570 (84%) directly stated their WTP for health gains, whereas 299 (16%) went through the bidding process. This finding is consistent with the results of our pilot survey. Most covariates showed standardised mean differences <0.1, indicating minimal imbalance between the elicitation groups (online supplemental figure S2). This suggests that observed differences in WTP by elicitation method are unlikely to be driven by systematic differences in participant characteristics.

### WTP estimates and their determinants

Table 2 presents the observed sample means by elicitation method and the estimated WTP values from generalised linear regression models. Direct elicitation yielded mean WTP values of 50 129 BTN (95% CI: 45 724 to 54 534) for 1 year of cancer symptom relief, 75 188 BTN (95% CI: 65 604 to 84 773) for 1 QALY and 169 490 BTN (95% CI: 154 866 to 184 113) for 5 QALYs. Mann-Whitney U tests confirmed that bidding elicitation systematically produced higher WTP values than direct elicitation across all scenarios (online supplemental table S1).

**Table 2:** WTP estimates for the three scenarios

	Observations	1 Year cancer Symptom-free	1 QALY	5 QALYs
Panel A: full sample				
Regression-adjusted WTP	1869	76 836 (71 397 to 82 275)	104 381 (96 405 to 112 357)	235 237 (218 674 to 251 800)
WTP by elicitation method				
Direct elicitation	1570	50 129 (45 724 to 54 534)	75 188 (65 604 to 84 773)	169 490 (154 866 to 184 113)
Bidding	299	208 054 (187 554 to 228 553)	251 037 (228 358 to 73 715)	562 866 (486 218 to 639 514)
Panel B: subsample (excluding null and inconsistent responses)				
Regression-adjusted WTP	1828	78 827 (73 289 to 84 365)	102 396 (96 030 to 108 763)	240 077 (223 136 to 257 019)
WTP by elicitation method				
Direct elicitation	1532	51 579 (47 038 to 56 121)	72 192 (65 995 to 78 389)	173 236 (158 301 to 188 172)
Bidding	296	211 290 (190 562 to 232 018)	250 135 (227 475 to 272 795)	567 807 (490 586 to 645 029)

WTP values are in Bhutanese Ngultrum. WTP estimates are derived from regression models in table 3, which control for demographics (age, sex and marital status), socioeconomic factors (education level, employment status, residential area, household size and annual income), health behaviours (smoking and drinking) and health status (EQ-5D-5L utility score <1, indicating imperfect health) and elicitation method.

QALY, quality-adjusted life year; WTP, willingness to pay.

Regression-adjusted WTP values were 76 836 BTN (0.26 times GDP per capita; 95% CI: 71 397 to 82 275) for 1 year of cancer symptom relief, 104 381 BTN (0.35 times GDP per capita; 95% CI: 96 405 to 1 12 357) for a 1-QALY gain and 235 237 BTN (0.78 times GDP per capita; 95% CI: 218 674 to 251 800) for a 5-QALY gain. Sensitivity analyses of a restricted sample, which excluded respondents with null and inconsistent responses, showed slight variations in estimates. In this subsample, the estimated WTP for 1 year of cancer symptom-free life was 78 827 BTN (0.26 times GDP per capita; 95% CI: 73 289 to 84 365). For a 1-QALY gain, it was 102 396 BTN (0.34 times GDP per capita; 95% CI: 96 030 to 108 763), and for a 5-QALY gain, it was 240 077 BTN (0.80 times GDP per capita; 95% CI: 223 136 to 25 709).

WTP determinants were assessed through associations with demographic, socioeconomic and health behaviour factors, adjusting for elicitation method (table 3). Among socioeconomic factors, household income emerged as a key determinant across all three scenarios. Each 100 000 BTN increase in per capita household income corresponded to a 10% (10 330 BTN) increase in WTP for a QALY gain, controlling for other variables. Education also served as a significant determinant, with secondary education and equivalent showing 15–26% higher WTP compared with no education. Those with tertiary education showed 36–48% higher WTP than those with no education. Other demographic and socioeconomic factors, including age, sex, marital status, working status, residential areas and health status, showed no significant association with WTP.

**Table 3:** Baseline analysis: WTP determinants

	1-year cancer symptom-free		1 QALY		5 QALYs	
	Coefficient (95% CI)	P value	Coefficient (95% CI)	P value	Coefficient (95% CI)	P value
Age	−0.0010 (−0.0061 to 0.0041)	0.70	−0.00055 (−0.0057 to 0.0046)	0.83	0.0046 (−0.0013 to 0.01)	0.12
Sex (Ref: male)						
Female	0.080 (−0.055 to 0.22)	0.25	0.14 (−0.000043 to 0.28)	0.050	0.028 (−0.11 to 0.16)	0.68
Marital status (Ref: never married)						
Married/living together	0.15 (−0.022 to 0.33)	0.086	0.082 (−0.084 to 0.25)	0.33	−0.13 (–0.34 to 0.078)	0.22
Divorced/separated/widowed	0.056 (−0.18 to 0.30)	0.65	−0.065 (−0.3 to 0.17)	0.58	−0.29 (−0.56 to −0.012)	0.041
Educational level (Ref: no education)						
Primary or below	0.073 (−0.081 to 0.23)	0.35	0.0043 (−0.15 to 0.16)	0.96	0.10 (−0.08 to 0.29)	0.27
Secondary or equivalent	0.23 (0.069 to 0.39)	< 0.01	0.23 (0.068 to 0.39)	< 0.01	0.14 (−0.039 to 0.32)	0.12
Tertiary	0.39 (0.15 to 0.64)	< 0.01	0.31 (0.086 to 0.54)	< 0.01	0.38 (0.12 to 0.63)	< 0.01
Working status (Ref: not working)						
Currently working	0.064 (−0.046 to 0.17)	0.25	0.098 (−0.021 to 0.22)	0.11	−0.016 (−0.14 to 0.11)	0.80
Per capita income (1000 BTN)	0.0013 (0.00056 to 0.0021)	< 0.01	0.0010 (0.00030 to 0.0017)	< 0.01	0.0011 (0.00047 to 0.0017)	< 0.01
Residential area (Ref: urban)						
Rural	0.046 (−0.094 to 0.19)	0.52	−0.088 (−0.23 to 0.055)	0.23	0.022 (−0.12 to 0.16)	0.76
Smoking status (Ref: current smoker)						
Ever smoker	0.074 (−0.17 to 0.31)	0.55	0.16 (−0.074 to 0.39)	0.18	0.097 (−0.14 to 0.33)	0.41
Non-smoker	0.078 (−0.14 to 0.3)	0.48	0.24 (0.023 to 0.45)	0.03	0.18 (−0.028 to 0.39)	0.089
Drinking frequency (Ref: not drinking)						
Frequent drinking	0.048 (−0.15 to 0.24)	0.63	0.19 (−0.014 to 0.4)	0.067	−0.0066 (−0.17 to 0.16)	0.94
Occasional drinking	0.0068 (−0.13 to 0.14)	0.92	0.076 (−0.069 to 0.22)	0.30	0.11 (−0.044 to 0.27)	0.16
Health status (Ref: perfect health)						
Imperfect health	−0.081 (−0.20 to 0.034)	0.17	−0.11 (−0.22 to 0.012)	0.079	−0.065 (−0.18 to 0.055)	0.29
Elicitation via bidding	1.3 (1.2 to 1.5)	<0.01	1.2 (1.1 to 1.3)	<0.01	1.1 (0.93 to 1.2)	<0.01
Intercept	10 (9.8 to 11)	< 0.01	11 (10 to11)	< 0.01	12 (11 to 12)	< 0.01
District	Yes		Yes		Yes	
Observations	1868		1868		1868	

Notes: results are rounded to two significant digits. Due to space constraints, detailed district-specific estimates are not included. Predicted district-specific WTP means, based on the same regression, are provided in online supplemental table S3Supplementary Table S3.

BTN, Bhutanese Ngultrum; QALY, quality-adjusted life year; WTP, willingness to pay.

Regarding health behaviours, non-smokers, including former smokers, consistently exhibited higher WTP than current smokers across all scenarios, although not all differences were statistically significant. In the 1-QALY scenario, non-smokers had a 27% higher WTP compared with current smokers (107 811 vs 85 223 BTN). Alcohol consumption patterns also showed some influence, with frequent drinkers expressing 21% higher WTP than non-drinkers in the past 12 months (120 429 vs 99 210 BTN), although this difference was only marginally significant (p<0.1). Additional sensitivity analyses in online supplemental table S2 further validated these findings. When excluding null and inconsistent responses, the associations between socioeconomic factors and WTP remained robust. The income effect slightly increased to 12% per 100 000 BTN, while other associations maintained similar magnitudes of effect.

### Geographic variation

Beyond individual characteristics, we examined variations in WTP through district-fixed effects (online supplemental table S3 and figure S3). The highest WTP values in the 1-QALY scenario (from 138 972 BTN to 275 596 BTN) were found in Trongsa, Zhemgang, Monggar, Gasa and Trashigang. In contrast, the lowest WTP values (from 66 043 BTN to 76 734 BTN) were in Tsirang, Thimphu, Samdrup Jongkhar, Sarpang and Dagana, with the capital district Thimphu showing a WTP of 67 343 BTN.

To quantify the relative importance of these geographic variations, we conducted a complementary multilevel analysis. The results suggest that geographic location had a minimal influence on overall WTP variability. In the random effects component of the analysis, ICCs from null models (without any predictors) showed that district-level differences explained only 2–6% of WTP variability across all scenarios. Even after controlling for individual characteristics and elicitation methods, ICCs remained below 10%, with values as low as 2.1% in the 1-QALY scenario (table 4). The fixed effects component further confirmed our primary findings from table 3, showing that individual characteristics, particularly income and education, were the significant determinants of WTP.

**Table 4:** Multilevel analysis: WTP variability

	1-year cancer symptom-free		1 QALY		5 QALYs	
	Null model	Full model	Null model	Full model	Null model	Full model
**Fixed effects**						
(Intercept)	11 (10 to 11)	10 (10 to 11)	12 (11 to 12)	11 (10 to 11)	12 (11 to 12)	12 (11 to 12)
Age		−0.00030 (−0.0060 to 0.0050)		−0.0010 (−0.0080 to 0.0060)		0.0050 (−0.0010 to 0.011)
Sex (Ref: male)						
Female		0.067 (−0.073 to 0.21)		0.12 (−0.047 to 0.30)		0.024 (−0.12 to 0.17)
Marital status (Ref: never married)						
Married/living together		0.13 (−0.061 to 0.32)		0.105 (−0.131 to 0.340)		−0.12 (−0.32 to 0.082)
Divorced/separated/widowed		0.044 (−0.23 to 0.32)		−0.042 (−0.38 to 0.29)		−0.28 (−0.57 to 0.0040)
Educational level (Ref: no education)						
Primary or below		0.068 (−0.11 to 0.24)		0.021 (−0.20 to 0.24)		0.12 (−0.072 to 0.30)
Secondary or equivalent		0.25 (0.081 to 0.43)		0.26 (0.050 to 0.47)		0.17 (–0.014 to 0.35)
Tertiary		0.39 (0.13 to 0.64)		0.30 (−0.019 to 0.62)		0.37 (0.10 to 0.65)
Working status (Ref: not working)						
Currently working		0.091 (−0.036 to 0.22)		0.13 (−0.029 to 0.28)		0.0033 (−0.13 to 0.14)
Per capita income (1000 BTN)		0.0012 (0.00050 to 0.0018)		0.00090 (0.00010 to 0.0017)		0.0010 (0.00030 to 0.0017)
Residential area (Ref: urban)						
Rural		0.024 (−0.12 to 0.17)		−0.096 (−0.27 to 0.076)		0.0030 (−0.15 to 0.15)
Smoking status (Ref: current smoker)						
Ever smoker		0.029 (−0.22 to 0.28)		0.12 (−0.180 to 0.428)		0.063 (−0.20 to 0.32)
Non-smoker		0.041 (−0.17 to 0.26)		0.24 (−0.023 to 0.50)		0.16 (−0.063 to 0.39)
Drinking frequency (Ref: not drinking)						
Frequent drinking		0.050 (−0.12 to 0.22)		0.20 (–0.0050 to 0.41)		−0.0030 (–0.18 to 0.17)
Occasional drinking		−0.010 (−0.16 to 0.14)		0.070 (−0.11 to 0.25)		0.095 (−0.060 to 0.25)
Health status (Ref: perfect health)						
Imperfect health		−0.059 (−0.18 to 0.062)		−0.076 (−0.23 to 0.072)		−0.055 (−0.18 to 0.072)
Elicitation via bidding		1.3 (1.2 to 1.5)		1.2 (1.0 to 1.4)		1.1 (0.91 to 1.3)
**Random effects**						
Variance (district)	0.24	0.25	0.15	0.15	0.29	0.31
Variance (individual)	4.1	3.3	8.6	6.9	4.3	4.0
ICC	0.055	0.070	0.017	0.021	0.063	0.072
N	1823	1822	1844	1843	1844	1843

Notes: Results are presented as coefficients with 95% CI. The null model excludes predictors, while the full model includes them. The estimates are rounded to two significant digits.

BTN, Bhutanese Ngultrum; ICC, intraclass correlation coefficient; QALY, quality-adjusted life year; Ref, reference; WTP, willingness to pay.

## Discussion

This study is the first to analyse WTP for health interventions in Bhutan, leveraging data from a comprehensive and nationally representative health survey. We systematically explore WTP across various demographic, socioeconomic, behavioural and geographic groups by linking WTP data with an extensive range of individual and household-level variables. These findings provide insights that inform health policy and decision-making.

Our analyses revealed variations in WTP across different scenarios. WTP estimates ranged from 76 836 BTN for an additional year free of cancer symptoms to 104 381 BTN for a 1-QALY gain and up to 235 237 BTN for a 5-QALY gain. However, these values must be interpreted in light of methodological differences. WTP values from the bidding process were 2–2.7 times higher than those from direct elicitation, consistent with well-documented anchoring effects in contingent valuation studies.^15^,^17^ Moreover, since elicitation methods were assigned based on difficulties with direct elicitation, individuals with higher underlying WTP may have been disproportionately assigned to the bidding format. This assignment pattern may have introduced upward bias in societal WTP estimates.

It can be noticed that WTP did not increase proportionately with the magnitude of the benefit (ie, QALYs). This non-linear relationship may be due to diminishing marginal utility, where the first QALY is perceived as more critical or life-changing than subsequent QALYs. Another possible explanation is the ceiling effect, where WTP reaches a threshold beyond which it no longer increases, even as health benefit grows.^18^ These findings are consistent with results from countries such as Japan, the UK, Denmark and Canada, which have shown diminishing returns on WTP for health benefits.^19^,^21^ While the discount rates, which reflect the preference for immediate benefits over future ones, could also influence WTP, they are unlikely to be the primary factor in this context. Given that 5 QALYs elicit only twice the WTP of 1 QALY, we would need to assume an implausibly high discount rate of over 25%. This far exceeds the 3–6% rates typically used in health economics studies.^22^

Household income was a key determinant of WTP, which is consistent with previous studies.^23^,^25^ Higher-income households, particularly those with members in managerial and clerical jobs, expressed higher WTP than lower-income households. However, the proportion of income allocated to health interventions varied and did not consistently correlate with income levels. For instance, some higher-income households allocated 50% of their income to health interventions, while lower-income households allocated up to 80% (online supplemental figure S4). This suggests that health prioritisation among households may not be solely income-driven but influenced by other factors, which require more data and further investigation.

Education level was another significant determinant of WTP. Individuals with secondary education or higher exhibited greater WTP for health interventions than those with lower education levels. This trend may be due to better awareness of health benefits, a greater understanding of health interventions or a higher perceived value of health among more educated individuals.^26^ When WTP responses vary due to differences in understanding rather than true differences in valuation or ability to pay, these financing decisions become less reliable. Our findings suggest that targeted educational programmes could help standardise knowledge across diverse groups. By providing a common understanding of the value and importance of healthcare services, these programmes could make WTP responses better reflect actual needs rather than varying levels of health literacy. Such reliable WTP data would support the development of scalable and sustainable healthcare financing models.

Health behaviours showed mixed patterns in WTP across substance use. Non-smokers, including former smokers, consistently expressed higher WTP than smokers. This pattern exists alongside Bhutan’s stringent tobacco control measures (sales bans, public smoking restrictions and high taxation) aimed at reducing smoking-related health risks. In contrast, the relationship between alcohol consumption and WTP was less consistent. While frequent drinkers exhibited a higher WTP than non-drinkers in the 1-QALY scenario, this pattern did not persist across other scenarios. This variation exists in a cultural context where alcohol is integrated into daily rituals and ceremonies.^27^

Multilevel analysis revealed that WTP variations were primarily driven by individual factors such as education or economic status, with minimal influence from geographic location. This finding supports implementing a national CET rather than a regional one. However, the socioeconomic gradient in WTP presents challenges for health system financing: setting CETs based on average WTP could drive up thresholds due to consistently higher values from wealthier, more educated individuals. This could strain limited public resources by making more expensive technologies appear cost-effective and thus eligible for public funding.

This challenge is particularly relevant for Bhutan’s public health sector, where most health services including medical expenses at designated hospitals abroad are provided through the benefit package. Previous economic evaluations in Bhutan, such as for HPV and pneumococcal vaccines, lack locally tailored threshold and used WHO-recommended 1 time GDP per capita or 0.5 times GDP per capita based on evidence from other countries.^6 28^ Our context-specific evidence offers more relevant guidance for setting appropriate thresholds. For services such as international referrals necessary for patient care, fully funding them could strain public resources. Policymakers may need to carefully balance coverage with sustainable resource allocation, for example, by covering essential medical costs rather than full expenses (eg, transportation costs related to international referral services) through the public healthcare system.

The socioeconomic gradient in WTP also has implications for pricing strategies for Bhutan’s emerging fee-based services. While healthcare services remain predominantly public, fee-based options are available through private diagnostic centres and after-hours services at public facilities.^8 29^ Determining pricing for these services lacks an empirical foundation, and WTP estimates could guide these evidence-based decisions. However, the higher values from wealthier groups suggest potential access barriers. Wherever appropriate, targeted subsidies or partial public involvement may be necessary to ensure equitable access to these services.

While WTP helps capture societal preferences for CET settings, it may not adequately represent actual resource constraints and healthcare costs, especially in Bhutan’s publicly funded health system. To ensure threshold reasonability and efficient resource allocation, supply-side evidence based on the marginal productivity of healthcare spending is needed to assess opportunity costs of service displacement. Future studies should incorporate a supply-side approach to better inform CET settings in Bhutan.^30^

Although this study adds to the limited literature on WTP in Bhutan and other LMICs, several limitations should be acknowledged. First, as part of an extensive national survey, the WTP survey was constrained in scope and depth. Unlike dedicated WTP surveys that explore multiple scenarios and diseases, our WTP section was brief, focusing on a few key scenarios due to the extensive number of questions in other sections. Moreover, the survey design did not systematically record which elicitation method was used for each respondent. While enumerators were trained in both approaches, we had to classify methods post-hoc based on response patterns, which introduces classification errors. These scope and methodological limitations may have affected the precision and comprehensiveness of our results.

Second, the context and framing of the scenario can affect the validity and consistency of the estimated CET. The hypothetical nature of our scenario setting may have influenced participants’ responses, potentially leading to over- or underestimation of true WTP. For instance, in the first scenario, we used a general framing that allowed respondents to draw on their understanding of cancer. While this approach made the scenario more relatable to participants, it may have introduced variability in how they conceptualised and valued the described health state.

Third, the use of two different elicitation methods introduces bias. Respondents who struggled with direct elicitation were guided through the bidding format. This conditional assignment resulted in systematically higher WTP values for those using bidding methods. Even when controlling for elicitation methods, WTP estimates should be interpreted with caution. Future studies should consider incorporating calibration techniques to adjust for methodological differences and improve the precision of WTP estimates.

Lastly, WTP values can be shaped by cultural and socioeconomic contexts, which limits the generalisability of these findings to other settings.

## Conclusion

This study estimated WTP for health improvements across disease scenarios through a population-based survey in Bhutan. The findings provide insights into the socioeconomic and behavioural determinants of WTP. Limited between-district variations in WTP suggest the feasibility of a national CET. However, within-district variations highlight the need to balance efficiency using individual factors with equity concerns in CET design. These findings offer valuable guidance for Bhutan’s healthcare resource allocation and serve as a reference for other LMICs seeking to use WTP per QALY to inform resource allocation in their healthcare systems.

## Supplementary material

10.1136/bmjgh-2025-019098 online supplemental file 1

## Data Availability

Data may be obtained from a third party and are not publicly available.
