# Supplementary material for: Valuing health across groups: a cross-sectional population-based willingness-to-pay survey in Bhutan
Source: BMJ Glob Health. 2025 Aug 21;10(8):e019098. doi: 10.1136/bmjgh-2025-019098 (PMC12374658; doi:10.1136/bmjgh-2025-019098)
Supplement: online supplemental file 1 [file bmjgh-10-8-s001.pdf]

APPENDIX

A. Survey Questionnaire

SECTION H: Health Status (EQ-5D-5L)

By placing a tick in one box in each group below, please indicate which statements best describe your own health state today.

Q 2.1 Mobility

I have no problems in walking about

2 I have slight problems in walking about

3 I have moderate problems in walking about

4 I have severe problems in walking about

5 I am unable to walk about

Q 2.2 Self-Care

1 I have no problems washing or dressing myself

2 I have slight problems washing or dressing myself

3 I have moderate problems washing or dressing myself

4 I have severe problems washing or dressing myself

5 I am unable to wash or dress myself

Q 2.3 Usual Activities *(e.g. work, study, housework, family or leisure activities)*

1 I have no problems doing my usual activities

2 I have slight problems doing my usual activities

3 I have moderate problems doing my usual activities

4 I have severe problems doing my usual activities

5 I am unable to do my usual activities

Q 2.4 Pain/Discomfort

1 I have no pain or discomfort

2 I have slight pain or discomfort

3 I have moderate pain or discomfort

4 I have severe pain or discomfort

5 I have extreme pain or discomfort

Q 2.5 Anxiety/Depression

1 I am not anxious or depressed

2 I am slightly anxious or depressed

3 I am moderately anxious or depressed

4 I am severely anxious or depressed

5 I am extremely anxious or depressed

We would like to know how good or bad your health is TODAY. This scale is numbered from 0 to 100.

100 means the best health you can imagine. 0 means the worst health you can imagine.

- Mark an X on the scale to indicate how your health is TODAY
- Now, please write the number you marked on the scale in the box below.

YOUR HEALTH TODAY =

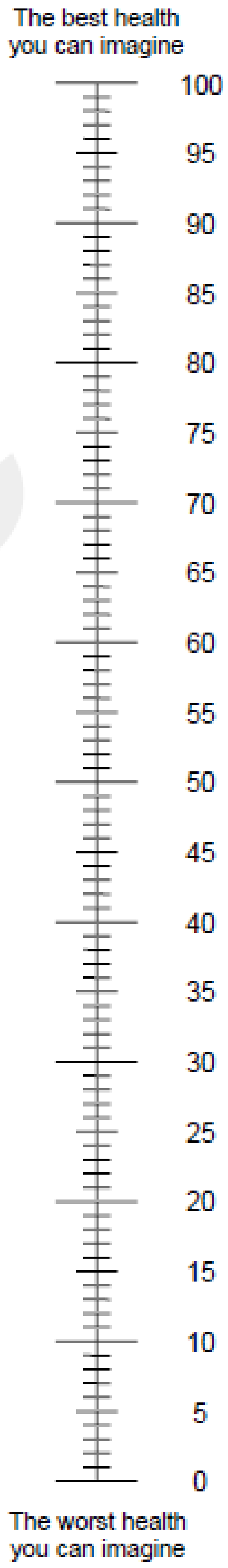

Source: adapted from the EuroQol Group: www.euroqol.org

SECTION H1: Willingness to Pay (WTP)

The enumerator reads to the respondent:

Now, I have some questions regarding your willingness to *pay (WTP) for health.*

*Note to Enumerator: If a respondent is unable to provide a specific amount, you will refer to the table (WTP card/Showcard) and assist him/her with a starting value of 30,000 Nu for questions H1 and H2 for one-year QALY and 70,000 Nu for question H3 for 5 years QALY.

Willing to pay card (Showcard)

Payment (BTN)/month	**Response (√)**	Payment (BTN)/month	**Response (√)**
**0**	❑ Yes ❑ No >>***GO TO H4***	**40,000**	❑ Yes ❑ No
**5,000**	❑ Yes ❑ No	**45,000**	❑ Yes ❑ No
**10,000**	❑ Yes ❑ No	**50,000**	❑ Yes ❑ No
**15,000**	❑ Yes ❑ No	**55,000**	❑ Yes ❑ No
**20,000**	❑ Yes ❑ No	**60,000**	❑ Yes ❑ No
**25,000**	❑ Yes ❑ No	**65,000**	❑ Yes ❑ No
**26,000**	❑ Yes ❑ No	**70,000**	❑ Yes ❑ No
**27,000**	❑ Yes ❑ No	**75,000**	❑ Yes ❑ No
**28,000**	❑ Yes ❑ No	**80,000**	❑ Yes ❑ No
**29,000**	❑ Yes ❑ No	**85,000**	❑ Yes ❑ No
**30,000**	❑ Yes ❑ No	**>90,000**	❑ Yes ❑ No

H1. WTP for one additional year of perfect health

Scenario 1*: Imagine that you are suffering from* ***Cancer*** *that* <u>*immediately threatens your life*</u>*. Now, please assume that we have a* ***new drug*** *that will provide relief from cancer symptoms or side effects from cancer treatments for a duration of ONE year. If the national health system does not fund the new drug and you must pay by yourself.*

How much are you willing to pay for the new drug? ______________BTN/month*

[Please show the WTP card to determine the maximum amount and then report it here]

H2. WTP for one additional year of perfect health

Scenario 2: *Imagine that you are* _stricken with a serious illness that immediately threatens your life_. *Now, please assume that we have a* ***new treatment*** *that allows you* to *return* to ***one-year*** *perfect health state (no pain, perfect mobility*, *total autonomy for personal care and daily activities, and absence of anxiety or depression). Imagine that the effect lasts only while you are taking it, and it has no side effects. If the national health system does not fund the new treatment and you must pay by yourself.*

How much are you willing to pay for the new treatment? ______________BTN/month*

[Please show the WTP card to determine the maximum amount and then report it here]

H3. WTP for five additional years of perfect health

Scenario 3*:* Imagine that you are stricken with a serious illness that immediately threatens your life. *Now, please assume that we have a* ***new treatment*** *that allows you to return to* ***a 5-year perfect*** *health state (no pain, perfect mobility, total autonomy for personal care and daily activities, and absence of anxiety or depression). Imagine that the effect lasts only while you are taking it, and it has no side effects. If the national health system does not fund the new treatment and you must pay by yourself.*

How much are you willing to pay for the new treatment? ______________BTN/month*

[Please show the WTP card to determine the maximum amount and then report it here]

Note: If the respondent reports WTP =0 in H1, H2, or H3, please ask question H4.

H4. Indicate the main reason why you are not willing to pay for an improvement in the quality of public healthcare services

(multi-select)

01 It is the government's responsibility to allocate more resources to healthcare services

02 My household cannot afford to make a contribution

03 It is our right to get the best quality health services without making a contribution

04 Only financially comfortable people should pay the contributions

05 I prefer other ways of making contributions

06 We do not intend to use public healthcare facilities in the future

07 I am not interested in these quality improvements

08 I do not wish to respond to a hypothetical scenario

96 Other (Please specify ……………………………………………)

98 Don’t know

Note: If the respondent reports WTP >0 in H1, H2, or H3, please ask question H5.

H5. Where (source) does this money come from?

(multi-select)

01 Regular income

02 Household saving

03 Sell of personal belongings (Jewelry, gadgets, collections, clothes)

04 Sell of Livestock

05 Sell of Agricultural/Tree product/Tree

06 Sell of permanent assets (land, orchard, building, shares, plants/factories, machinery, vehicles)

07 Mortgage of Assets/Land

08 I do not wish to respond to a hypothetical scenario

09 Borrowed from Friends/Relatives/Office

10 Borrowed from Money Lender

11 Assistance from friends & relatives

96 Other (Please specify ……………………………………………)

98 Don’t know

B. Tables

Table S1. Mann-Whitney U Test Statistics for WTP Differences Between Elicitation Methods

	Direct elicitation		Bidding method		Mann-Whitney U Test	
Scenario	Mean	Median	Mean	Median	z	p
1-Year Cancer Symptom-free	50,129	208,055	208,053	120,000	-22.208	0.000
1 QALY	75,188	45,000	251,037	180,000	-20.597	0.000
5 QALYs	169,490	80,000	562,866	360,000	-18.203	0.000

Table S2. Sensitivity Analysis: WTP Determinants

	1-Year Cancer Symptom-free		1 QALY		5 QALYs	
	Coeff. (95% CI)	P-value	Coeff. (95% CI)	P-value	Coeff. (95% CI)	P-value
Age	0.00012 (-0.0049 – 0.0051)	0.96	0.00069 (-0.0044 – 0.0058)	0.79	0.0052 (-0.00059 – 0.011)	0.078
Sex (Ref: Male)						
Female	0.057 (-0.079 – 0.19)	0.41	0.021 (-0.11 – 0.15)	0.74	0.025 (-0.11 – 0.16)	0.72
Marital status (Ref: Never married)						
Married/Living together	0.14 (-0.033 – 0.32)	0.11	0.073 (-0.09 – 0.24)	0.38	-0.13 (-0.34 – 0.078)	0.22
Divorced/Separated/Widowed	0.045 (-0.2 – 0.28)	0.72	-0.095 (-0.32 – 0.13)	0.41	-0.3 (-0.58 – -0.025)	0.033
Educational level (Ref: No education)						
Primary or below	0.06 (-0.095 – 0.21)	0.45	0.039 (-0.12 – 0.2)	0.63	0.11 (-0.076 – 0.29)	0.25
Secondary or equivalent	0.25 (0.093 – 0.42)	<0.01	0.21 (0.047 – 0.37)	0.011	0.17 (-0.014 – 0.35)	0.071
Tertiary	0.38 (0.13 – 0.62)	<0.01	0.31 (0.083 – 0.53)	<0.01	0.38 (0.13 – 0.63)	<0.01
Working status (Ref: Not working)						
Currently working	0.067 (-0.044 – 0.18)	0.24	0.019 (-0.09 – 0.13)	0.73	-0.013 (-0.14 – 0.11)	0.84
Per capita income (1000 BTN)	0.0013 (0.00051 – 0.002)	<0.01	0.0012 (0.0005 – 0.0019)	<0.01	0.0011 (0.00047 – 0.0017)	<0.01
Residential area (Ref: Urban)						
Rural	0.02 (-0.12 – 0.16)	0.79	-0.006 (-0.14 – 0.12)	0.93	-0.003 (-0.14 – 0.13)	0.97
Smoking status (Ref: Current smoker)						
Ever smoker	0.024 (-0.22 – 0.27)	0.85	0.14 (-0.091 – 0.36)	0.24	0.078 (-0.15 – 0.31)	0.51
Non-smoker	0.039 (-0.18 – 0.26)	0.73	0.22 (0.017 – 0.43)	0.034	0.16 (-0.051 – 0.37)	0.14
Drinking frequency (Ref: Not drinking)						
Frequent drinking	0.037 (-0.16 – 0.23)	0.71	0.02 (-0.14 – 0.18)	0.81	-0.0015 (-0.17 – 0.16)	0.99
Occasional drinking	-0.0094 (-0.14 – 0.12)	0.89	0.09 (-0.053 – 0.23)	0.22	0.096 (-0.058 – 0.25)	0.22
Health status (Ref: Perfect health)						
Imperfect health	-0.062 (-0.18 – 0.053)	0.29	-0.052 (-0.16 – 0.06)	0.36	-0.053 (-0.17 – 0.066)	0.38
Intercept	10 (90.8 – 11)	<0.01	11 (10 – 11)	<0.01	12 (11 – 12)	<0.01
Bidding elicitation	10.3 (10.2 – 10.4)	<0.01	10.2 (10.1 – 10.3)	<0.01	10.1 (0.92 – 10.2)	<0.01
District	Yes		Yes		Yes	
Observations	1,806		1,827		1,827	

Note: The regressions use adjusted samples, which exclude null and inconsistent responses.

Table S3. District-Specific WTP Estimates

	1-year Cancer symptom-free	1 QALY	5 QALYs
	Predicted Mean (95 CI%)	Predicted Mean (95 CI%)	Predicted Mean (95 CI%)
Bumthang	66,932 (48,015–85,849)	128,212 (85,016–171,409)	220,391 (153,100–287,683)
Chhukha	71,589 (59,581–83,598)	110,710 (91,976–129,444)	250,010 (203,636–296,384)
Dagana	60,089 (45,907–74,271)	76,734 (52,622–100,846)	103,586 (72,675–134,498)
Gasa	94,657 (54,994–134,319)	140,141 (80,109–200,174)	241,860 (151,904–331,815)
Haa	72,524 (55,952–89,097)	106,926 (79,442–134,409)	246,383 (168,542–324,225)
Lhuentse	52,727 (37,346–68,109)	104,317 (67,841–140,793)	145,534 (83,718–207,350)
Monggar	140,028 (86,506–193,551)	149,117 (111,522–186,712)	458,154 (324,186–592,122)
Paro	69,779 (48,667–90,890)	83,559 (63,264–103,855)	161,776 (109,101–214,452)
Pema Gatshel	29,096 (21,304–36,888)	122,313 (–6,951–251,577)	92,745 (67,935–117,556)
Punakha	58,407 (44,231–72,584)	77,820 (58,529–97,111)	133,431 (97,704–169,158)
Samdrup Jongkhar	65,067 (46,967–83,167)	75,767 (56,881–94,652)	165,176 (131,274–199,078)
Samtse	62,061 (53,933–70,189)	86,919 (74,222–99,615)	154,564 (128,633–180,495)
Sarpang	70,328 (50,350–90,306)	76,319 (55,739–96,898)	174,980 (136,634–213,327)
Thimphu	49,488 (43,311–55,664)	67,343 (59,070–75,615)	162,612 (137,591–187,633)
Trashigang	121,070 (91,473–150,667)	138,972 (108,534–169,410)	338,733 (263,226–414,239)
Trashi Yangtse	130,525 (71,924–189,125)	126,851 (79,111–174,591)	459,793 (216,966–702,619)
Trongsa	128,664	275,596	617,167

	(91,488–165,841)	(216,914–334,277)	(467,583–766,751)
Tsirang	42,172<br>(32,991–51,352)	66,043<br>(51,966–80,121)	174,000<br>(133,154–214,846)
Wangdue Phodrang	92,452<br>(70,555–114,349)	107,416<br>(78,551–136,281)	316,565<br>(200,971–432,158)
Zhemgang	90,903<br>(61,851–119,955)	149,566<br>(45,042–254,091)	296,632<br>(141,498–451,766)

Note: The estimates are derived from the regressions presented in Table 3.

C. Figures

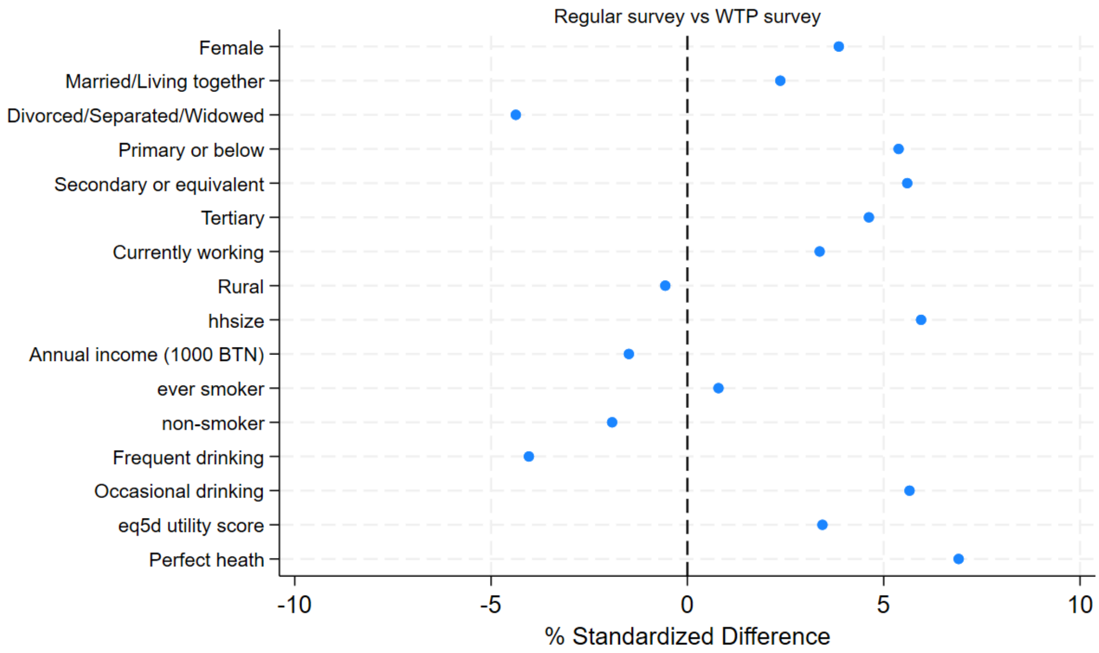

Figure S1. Standardized Mean Differences between Regular and WTP Survey Participants

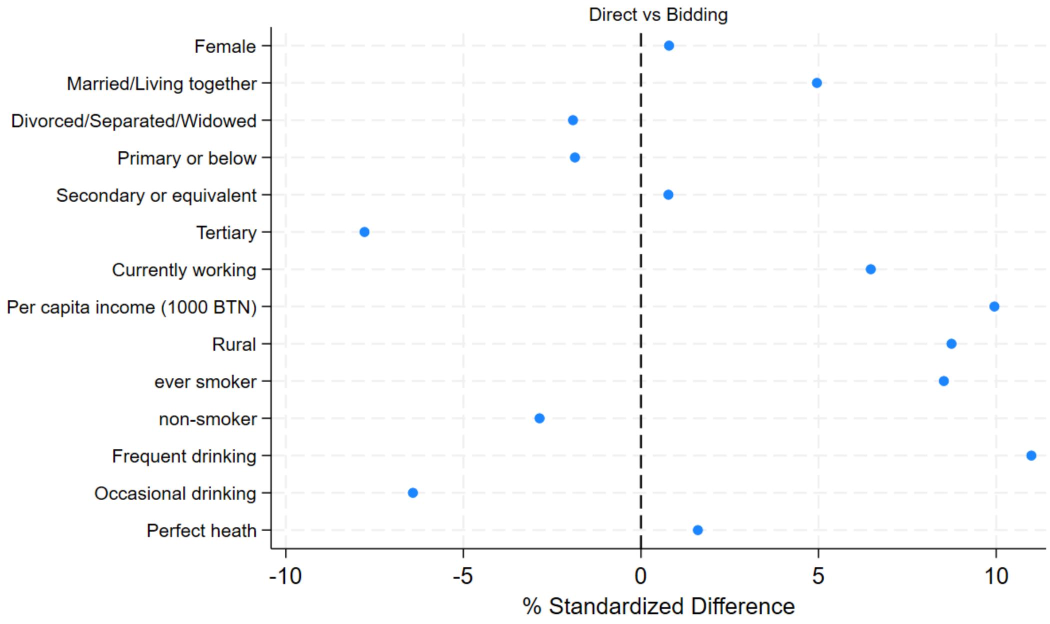

Figure S2. Standardized Mean Differences between Elicitation Groups

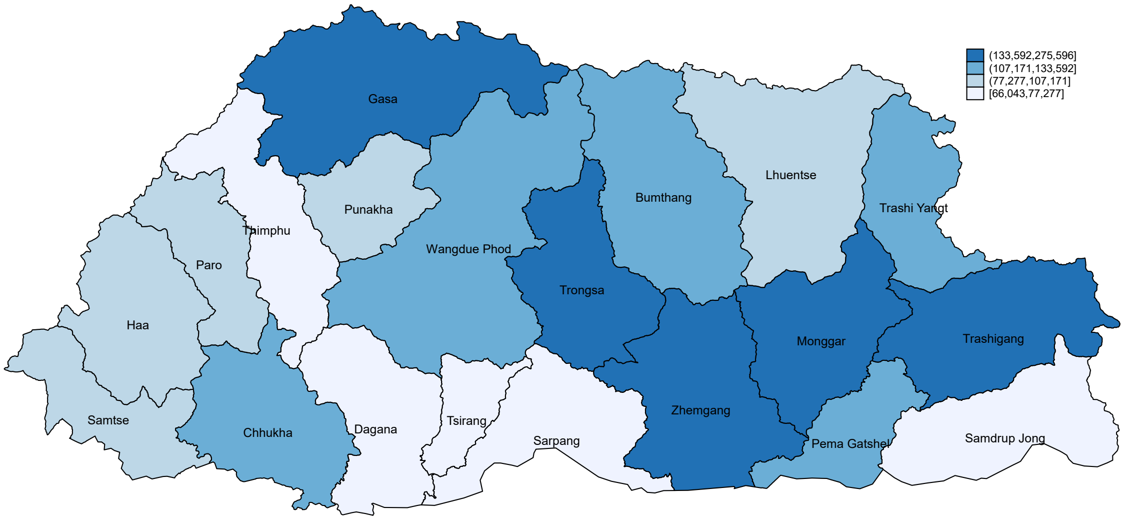

Note: The figure visualizes the WTP for 1-QALY health gain estimated at the district level (Supplementary Table S3).

Figure S3. WTP per QALY Across the District

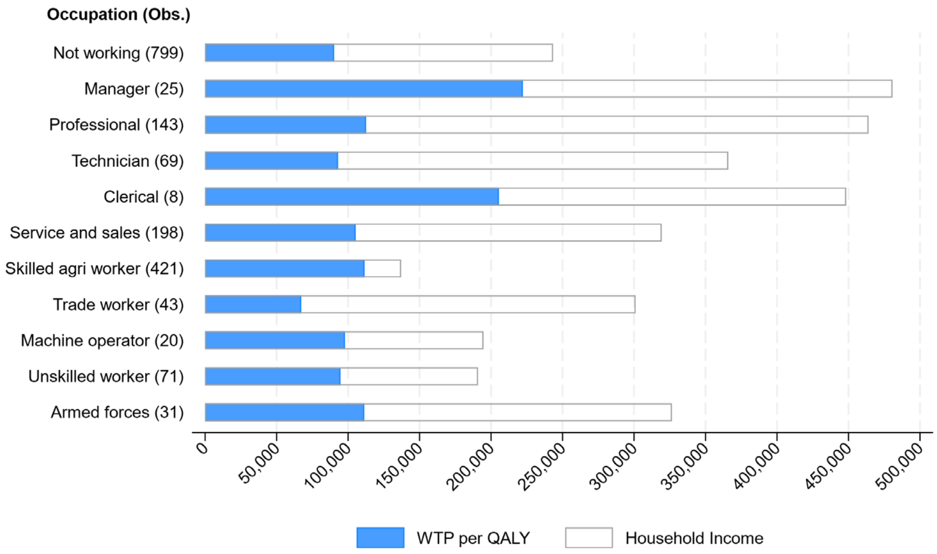

Figure S4. WTP per QALY and Household Income by Occupation
